# Mechanistic insights into Y-box binding protein-1 mediated regulation of lipid metabolism and oxidative stress in NAFLD via INHBE/TNF-β pathway

**DOI:** 10.17305/bb.2024.11249

**Published:** 2024-12-25

**Authors:** Zhi Ren, Rui Wang, Jun Wei, Zhenzeng Ma, Xiquan Ke

**Affiliations:** 1 The Gastroenterology Department, First Affiliated Hospital of Bengbu Medical University, Bengbu, Anhui, China; 2 The Oncology Department, First Affiliated Hospital of Bengbu Medical University, Bengbu, Anhui, China

**Keywords:** Y-box binding protein-1, YB1, lipid metabolism, nonalcoholic fatty liver disease, NAFLD, inhibin beta E, INHBE, TNF-beta signaling pathway

## Abstract

Nonalcoholic fatty liver disease (NAFLD) is a prevalent liver disorder that has emerged as a significant public health concern. This study aimed to investigate the mechanisms by which Y-box binding protein-1 (YB1) knockdown influences lipid metabolism and oxidative stress in palmitic acid (PA)-induced NAFLD LO2 cells. The expression of YB1 was analyzed using the GSE89632 dataset from the Gene Expression Omnibus (GEO) database. RNA sequencing was performed, followed by Gene Ontology (GO), Kyoto Encyclopedia of Genes and Genomes (KEGG) pathway analyses, and protein-protein interaction (PPI) network analyses to identify differentially expressed genes (DEGs). Quantitative real-time PCR (QRT-PCR), Western blotting, flow cytometry, and various biochemical assays were used to evaluate gene expression, lipid accumulation, and oxidative stress. Our results demonstrated that YB1 is highly expressed in NAFLD. RNA sequencing revealed 798 DEGs between the shCtrl and shYB1 groups, with 190 genes upregulated and 608 genes downregulated. Notably, we observed an increase in Inhibin beta E (INHBE) expression, while EGR1, GDF15, NUPR1, and FOSB were decreased in NAFLD LO2 cells. YB1 knockdown, particularly when combined with INHBE suppression, significantly enhanced cell viability, improved lipid metabolism, and reduced reactive oxygen species (ROS) accumulation and malondialdehyde (MDA) content. The downstream mechanism was primarily associated with TNF-β signaling. Specifically, we observed decreased levels of TGF-β1, p-Smad2, and p-Smad3 following YB1 and INHBE knockdown. Furthermore, INHBE overexpression reversed the beneficial effects induced by YB1 knockdown. In conclusion, YB1 knockdown improves lipid metabolism and reduces oxidative stress in NAFLD LO2 cells, largely through the INHBE/TNF-β signaling pathway. These findings provide valuable insights into novel therapeutic strategies for managing NAFLD.

## Introduction

Nonalcoholic fatty liver disease (NAFLD) is a prevalent and increasingly recognized liver condition that affects a substantial portion of the global adult population [1]. Defined by the accumulation of fat in liver cells in the absence of excessive alcohol consumption, NAFLD can progress to more severe forms, such as nonalcoholic steatohepatitis (NASH), which may lead to fibrosis, cirrhosis, and hepatocellular carcinoma [2]. The pathogenesis of NAFLD is commonly described by the two-hit hypothesis: the first hit involves abnormal triglyceride (TG) accumulation within hepatocytes, while the second hit involves the release of inflammatory mediators that drive liver damage, inflammation, and fibrosis [3, 4]. This progression highlights the need for a deeper understanding of NAFLD mechanisms, its interplay with metabolic disorders, and the development of effective therapeutic strategies to mitigate the global burden of liver disease. Y-box binding protein-1 (YB1), a member of the Cold Shock Domain protein superfamily, is a highly conserved DNA/RNA-binding protein located primarily in the cytoplasm and nucleus [5]. YB1 is associated with diverse biological functions, including gene expression regulation, stress responses, and cellular transformation [6]. Recent studies have implicated YB1 in various cancers, such as multiple myeloma, liver cancer, prostate cancer, lung cancer, and gastric cancer, where it influences tumorigenesis, progression, invasion, migration, drug resistance, and prognosis [7–11]. In liver pathology, YB1 has been linked to liver fibrosis through modulation of CXCL1 expression and liver-kidney interactions [12–14]. Additionally, emerging evidence suggests that YB1 regulates liver lipid metabolism via the Wnt/β-catenin signaling pathway, which is critical for maintaining cellular homeostasis and metabolic processes [15]. Despite these findings, the role of YB1 in NAFLD remains poorly understood. Exploring the interactions between YB1 and key metabolic and inflammatory pathways in NAFLD could provide valuable insights into disease mechanisms and identify potential therapeutic targets. This study aims to investigate the effects of YB1 knockdown on lipid metabolism and oxidative stress in palmitic acid (PA)-induced LO2 cells, a widely used model of NAFLD. Using molecular and biochemical assays, including RNA sequencing and pathway analysis, the research seeks to uncover the mechanisms through which YB1 influences NAFLD, with a particular focus on the inhibin beta E (INHBE)/TNF-β signaling pathway.

## Materials and methods

### Gene Expression Omnibus (GEO) database analysis

A search of the GEO database (accessible at https://www.ncbi.nlm.nih.gov/geo/) using the keyword “nonalcoholic fatty liver disease” identified the relevant human dataset GSE89632. This dataset includes 24 healthy control (HC) samples and 19 NAFLD samples. Gene expression profiles from GSE89632 were retrieved using the “GEOquery” package. Probe identifiers were mapped to gene symbols based on the manufacturer’s annotation files. For genes represented by multiple probes, the median expression value of all associated probes was calculated to address duplicates. Differential expression box plots for the gene “YB1,” comparing HC and NAFLD groups, were created using the ‘ggplot2’ package.

### Cell culture

LO2 cells (Procell, Wuhan, China), verified via short tandem repeat (STR) analysis, were cultured in DMEM medium (Gibco, USA) supplemented with 10% fetal bovine serum and 1% penicillin–streptomycin. The cells were maintained in an incubator at 37 ^∘^C with 5% CO_2_ and passaged when their growth density reached 80%–90%.

### *In vitro* NAFLD model

PA, sourced from MCE, USA, was dissolved in a 0.01 M NaOH solution and heated in a 75 ^∘^C water bath for 30 min to prepare a 20 mM PA solution. LO2 cells in the logarithmic growth phase were seeded in a six-well plate and subsequently treated with the PA solution. To establish the *in vitro* NAFLD model, the cells were exposed to 0.4 mM PA for 72 h.

### Cell transfection

YB1 shRNA, INHBE siRNA, and overexpression plasmids were designed by BIOMEDICAL (Anhui, China). LO2 cells were seeded in six-well plates and transfected with 100 pmol of RNA, which had been diluted in 250 µL of serum-free DMEM. Separately, 3 µL of NanoTrans reagent (BIOMEDICAL, Anhui, China) was diluted in another 250 µL of serum-free DMEM. The diluted RNA and NanoTrans reagent were combined and incubated at room temperature for 15 min to form the NanoTrans RNA complex. This complex was then evenly distributed into the culture medium. Eight hours post-transfection, the medium was replaced with fresh complete culture medium containing serum and antibiotics.

### RNA sequencing

After LO2 cells were transfected with shYB1 for 48 h, the culture medium was removed, and the cells were washed twice with PBS. TRIzol cell lysate was then added, and the samples were stored at −80 ^∘^C. Transcriptome sequencing analysis was subsequently performed by BioMarker Technology (Beijing, China). This process primarily involved pre-treatment of the original samples, reference genome alignment, and gene expression profiling analysis. In brief, total RNA was extracted from LO2 cells using TRIzol reagent. After quality control, the NEBNext Ultra RNA Library Prep Kit for Illumina (NEB, E7530) and NEBNext Multiplex Oligos for Illumina (NEB, E7500) were used to construct the cDNA library. The cDNA libraries were sequenced using an Illumina HiSeq™ sequencing platform. Gene expression levels were calculated using FPKM values (fragments per kilobase of exon per million fragments mapped) through Cufflinks software after mapping to the reference genome. Differentially expressed genes (DEGs) were identified using DESeq2, with criteria set at a *P* value < 0.05 and log2 fold change (log2FC) > 1. Upregulated genes were defined as those with log2FC > 1, while downregulated genes were defined as those with log2FC < −1. Based on the list of DEGs, Gene Ontology (GO), and Kyoto Encyclopedia of Genes and Genomes (KEGG) pathway enrichment analyses, as well as protein–protein interaction (PPI) network analyses, were conducted. The related and activated pathways were identified using Database for Annotation, Visualization and Integrated (DAVID).

### PPI network analysis, GO analysis, and KEGG pathway enrichment analysis for DEGs

PPI network analysis, GO analysis, and KEGG pathway enrichment analysis were performed using the DEGs identified (including INHBE) in the previous step via DAVID (version 6.8).

### CCK8 assay

Following digestion of LO2 cells, a cell suspension was prepared and inoculated into a 96-well plate at a concentration of 2 × 10^ImEquation2^ cells per well. The cells were pre-cultured in a constant temperature incubator for 12 h before being treated according to the respective experimental groups. Cell viability was assessed using the CCK-8 assay (BIOMEDICAL, Anhui, China), and the survival rate was determined by measuring the optical density at 450 nm.

### BODIPY staining

Lipid droplets in LO2 cells were detected using the BODIPY 493/503 Staining Kit (Beyotime, Shanghai, China). Following trypsin digestion, the cells were resuspended in PBS, washed, and centrifuged at 1000 × *g* for 5 min at room temperature. After discarding the supernatant, the cells underwent an additional PBS wash and were centrifuged at 400 × *g* for 5 min. The resulting cell pellet was then resuspended in 0.5 mL of staining solution and incubated in the dark at room temperature for 10 min. Fluorescence detection was performed using a flow cytometer.

### Oil Red O staining

Cells were fixed with 4% paraformaldehyde for 20 min, differentiated using 60% isopropanol for 3 min, and stained with freshly prepared oil red O staining solution for 20 min. Excess dye was removed, and the nuclei were counterstained with Mayer’s hematoxylin for 30 s. After rinsing with distilled water, the cells were observed and photographed under a microscope (Mshot, Guangzhou, China).

### TG, free fatty acid (FFA), and malondialdehyde (MDA) detection

TG levels in the cell lysate were measured using a TG assay kit (mlbio, Shanghai, China). FFA content in the cell lysate was determined with the Amplex Red Free Fatty Acid Assay Kit (Beyotime, Shanghai, China) following the manufacturer’s instructions. MDA levels in the cell lysate were quantified using the MDA assay kit (Solarbio, Beijing, China) according to the manufacturer’s protocol.

### Reactive oxygen species (ROS) detection

ROS were detected using 2′,7′-dichlorodihydrofluorescein diacetate (DCFH-DA; Solarbio, Beijing, China). Cells were cultured at a density of 5 × 10^ImEquation3^ cells/mL and pre-incubated for 1 h with or without 10 mM N-acetylcysteine (NAC). Following treatment with 150 µM Rg18 for 24 h, the cells were harvested, resuspended in pre-warmed PBS at 37 ^∘^C, and incubated with 20 µM DCFH-DA for 30 min at 37 ^∘^C. Fluorescence intensity was then measured using a flow cytometer (Beckman, USA).

### Quantitative real-time PCR (QRT-PCR)

Total RNA was extracted from each group using TRIzol reagent (Beyotime, Shanghai, China), and its purity and concentration were assessed using a micro-spectrophotometer. RNA was reverse-transcribed into cDNA under the following conditions: 42 ^∘^C for 15 min, 85 ^∘^C for 2 min, and 4 ^∘^C for 5 min. QRT-PCR was performed with the SYBR Green II premix (Takara, Japan) on a real-time PCR instrument using the following cycling conditions: 95 ^∘^C for 10 min, followed by 40 cycles of 95 ^∘^C for 15 s and 60 ^∘^C for 45 s. Dissociation curves were generated at 95 ^∘^C for 15 s, 60 ^∘^C for 60 s, and 95 ^∘^C for 15 s.

The GAPDH gene was used as an internal control, and relative mRNA expression levels were calculated using the 2^−ΔΔCt^ method. Primer sequences are provided in Table S1.

### Western blotting

LO2 cells from each experimental group were lysed using Radio Immunoprecipitation Assay (RIPA) buffer (Solarbio, Beijing, China). Protein concentrations were measured using a BCA assay (Solarbio, Beijing, China). The lysates were combined with 5× protein loading buffer and denatured in a 99 ^∘^C water bath for 10 min. For each sample, 30 µg of protein was separated by SDS-PAGE and transferred onto a PVDF membrane.The membrane was blocked with 5% skim milk (Solarbio, Beijing, China) for 1 h and incubated overnight at 4 ^∘^C with the following primary antibodies: INHBE (1:1000, ab103167, Abcam, UK), TGF-β1 (1:1000, Proteintech, 21898-1-AP, Wuhan, China), SMAD2 (1:2000, Proteintech, 12570-1-AP, Wuhan, China), SMAD3 (1:2000, Proteintech, 66516-1-AP, Wuhan, China), p-SMAD2 (1:1000, Affinity, AF3449, Jiangsu, China), p-SMAD3 (1:1000, Affinity, AF3365, Jiangsu, China), and GAPDH (1:10000, Proteintech, 60004-1-Ig, Wuhan, China). Following primary antibody incubation, the membrane was washed three times with phosphate-buffered saline with Tween-20 (PBST) for 10 min each. It was then incubated at room temperature for 1 h with the appropriate HRP-conjugated secondary antibody (1:10000, abs20040, Shanghai, China), either goat anti-rabbit IgG-HRP or goat anti-mouse IgG-HRP. After three additional 10-min washes with PBST, the membrane was developed using an enhanced chemiluminescence (ECL) reagent (Proteintech, Wuhan, China). Protein band intensities were quantified using ImageJ software.

### Ethical statement

Serum samples were collected from 15 healthy volunteers (Control) and 15 NAFLD patients. All participants provided informed consent, and the study complied with the principles of the Declaration of Helsinki. The research protocol received approval from the Institutional Review Board of the First Affiliated Hospital of Bengbu Medical University (Approval Number: 2023447).

### Statistical analysis

Statistical analyses were conducted using GraphPad Prism 9 software (GraphPad Software, USA). Data are expressed as the mean ± standard deviation (SD). Differential expression box-and-whisker plots for “YB1” were generated using the Wilcoxon test. Correlation analysis was performed with Spearman’s rank correlation method using the R package “corrplot.” One-way ANOVA was utilized for comparisons among multiple groups, while the *t*-test was applied for comparisons between two groups. Each experiment was conducted independently at least three times, and all statistical tests were two-tailed. A *P* value of less than 0.05 was considered statistically significant.

**Figure 1.**
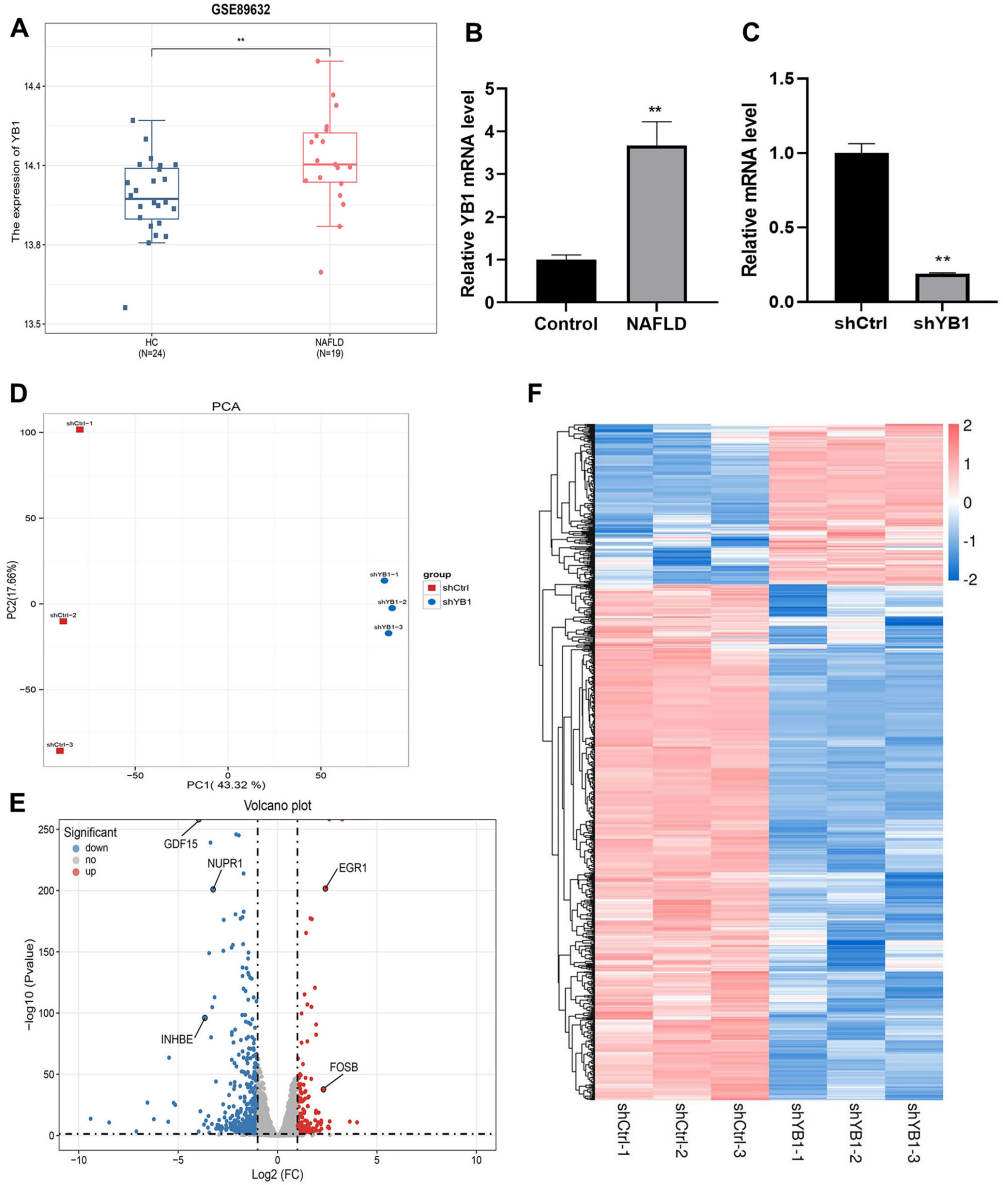
**DEGs were analyzed after YB1 knockdown in NAFLD model cells.** (A) YB1 was highly expressed in GSE89632 from GEO database; (B) YB1 was highly expressed in NAFLD cells; (C) QRT-PCR was used to detect the expression of YB1 after transfected with YB1 lentivirus; (D) PCA results presented two distinct populations could be clearly separated from each other in the shCtrl and the shYB1 groups; (E) Volcano plot of DEGs. The blue dots represented downregulated DEGs, the red dots represented upregulated DEGs, and the gray dots represented nondifferentially expressed genes; (F) Cluster analysis of DEGs in the shCtrl and shYB1 groups. NAFLD: Nonalcoholic fatty liver disease; YB1: Y-box binding protein-1; GEO: Gene Expression Omnibus; QRT-PCR: Quantitative real-time PCR; PCA: Principal component analysis; DEG: Differentially expressed gene.

## Results

### YB1 is highly expressed in NAFLD and DEGs were analyzed after YB1 knockdown in NAFLD model cells

**Figure 2.**
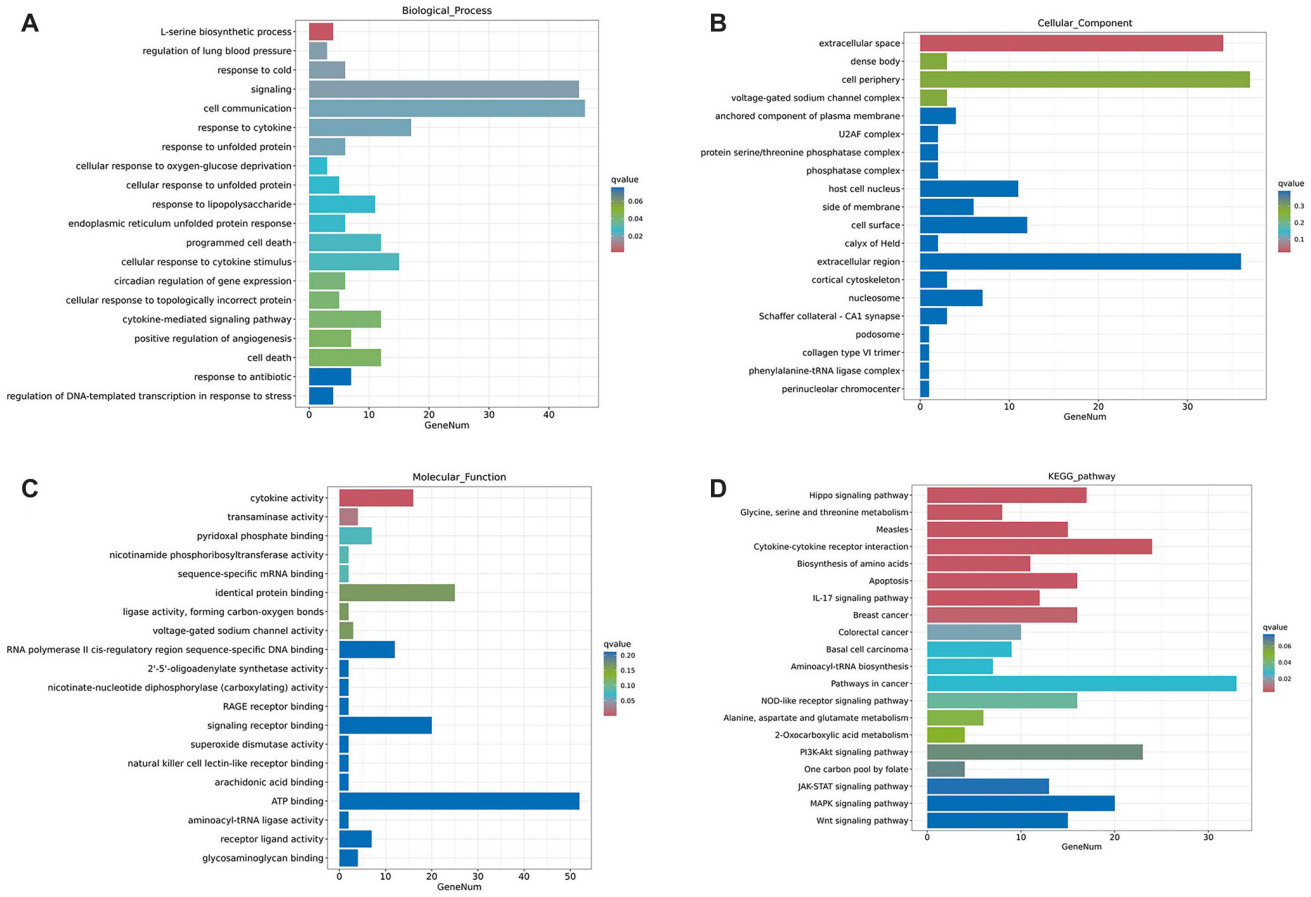
**GO and KEGG enrichment analysis for DEGs.** (A) GO for BP enrichment analysis; (B) GO for CC enrichment analysis; (C) GO for MF enrichment analysis; (D) KEGG enrichment analysis. GO: Gene Ontology; KEGG: Kyoto Encyclopedia of Genes and Genomes; DEG: Differentially expressed gene.

**Figure 3.**
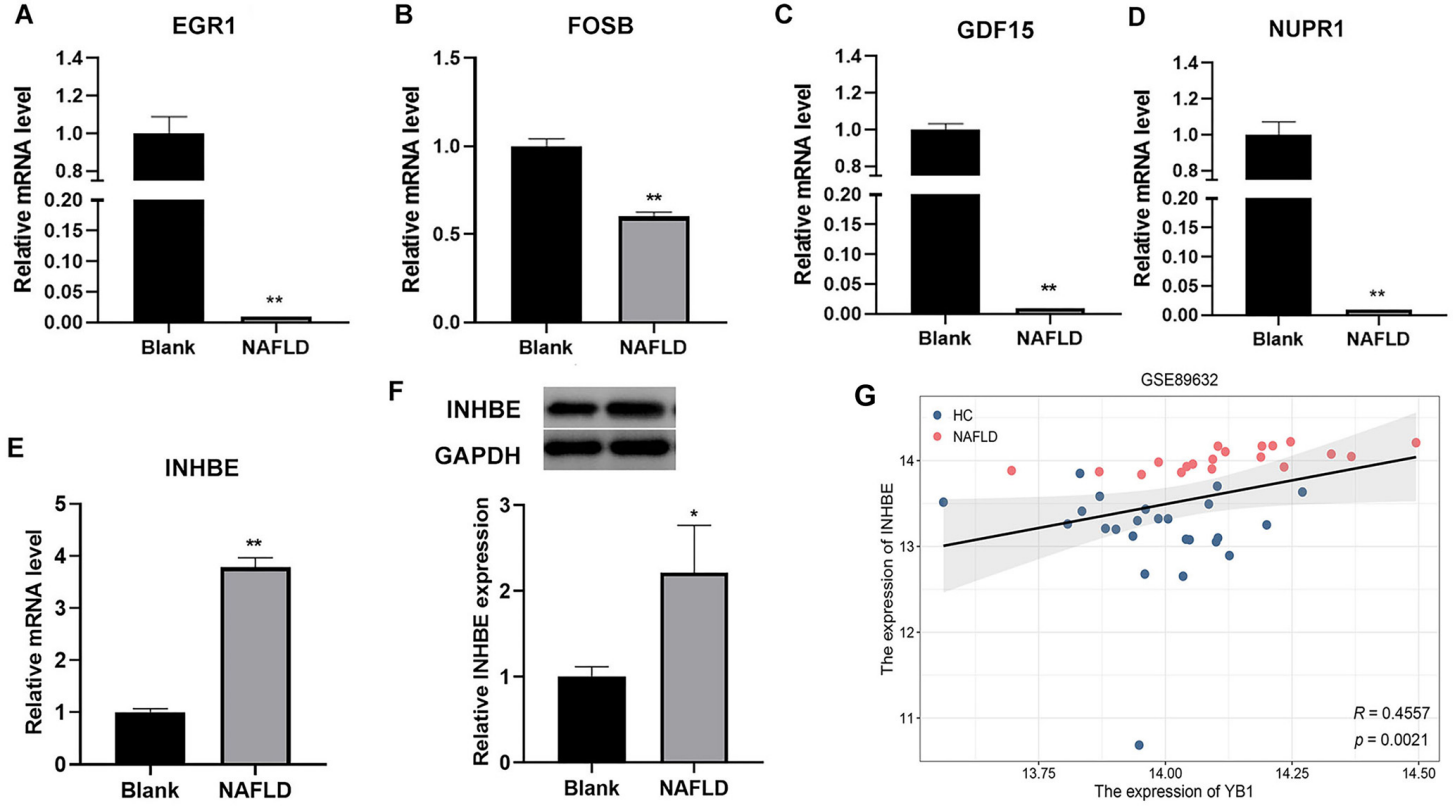
**The expression of five hub genes.** (A–E) QRT-PCR was used to detect the expression EGR1 (A), FOSB (B), GDF15 (C), NUPR1 (D), and INHBE (E); (F) Western blot was used to detect the expression of INHBE; (G) YB1 and INHBE showed a significant positive correlation in GSE89632 database. Compared to the blank group, *P < 0.05, **P < 0.01. QRT-PCR: Quantitative real-time PCR; INHBE: Inhibin beta E; YB1: Y-box binding protein-1.

To explore the role of YB1 in NAFLD, we selected dataset GSE89632 from the GEO database. The results showed that the expression level of YB1 was significantly higher in NAFLD samples compared to the HC group (Figure 1A, *P* < 0.01). Additionally, YB1 was highly expressed in a NAFLD cell model (Figure 1B, *P* < 0.01). To further investigate, we examined YB1 expression in the NAFLD model after YB1 knockdown. The results indicated a significant reduction in YB1 levels in the shYB1 group (Figure 1C, *P* < 0.01). Principal component analysis (PCA) revealed clear separation between the shCtrl and shYB1 groups, forming two distinct populations (Figure 1D). RNA sequencing identified 798 DEGs, with 190 genes upregulated and 608 downregulated (Figure 1E). Hierarchical clustering illustrated clear differences in gene expression between the two groups (Figure 1F). GO and KEGG functional enrichment analyses were conducted to categorize the DEGs. GO analysis showed that DEGs were primarily enriched in processes, such as the L-serine biosynthetic process, regulation of lung blood pressure, response to cold, cell signaling, cell communication, response to unfolded protein, cytokine activity, and transaminase activity (Figure 2A–2C). KEGG pathway analysis revealed significant enrichment in pathways, including Hippo signaling, glycine, serine, and threonine metabolism, cytokine–cytokine receptor interaction, biosynthesis of amino acids, apoptosis, IL-17 signaling, and pathways related to breast and colorectal cancers (Figure 2D). Based on *P* values and log2FC, five genes—EGR1 (*P* < 0.01, log2FC ═ 2.41), GDF15 (*P* ═ 0, log2FC ═ −3.96), INHBE (*P* < 0.01, log2FC ═ −3.66), NUPR1 (*P* < 0.01, log2FC ═ −3.24), and FOSB (*P* < 0.01, log2FC ═ 2.31)—were selected for further analysis. Expression analysis in normal LO2 cells and NAFLD model cells revealed that EGR1, GDF15, NUPR1, and FOSB were significantly downregulated in NAFLD cells, while INHBE was upregulated (Figure 3A–3E, *P* < 0.01). Western blot analysis further confirmed elevated INHBE expression in NAFLD cells (Figure 3F, *P* < 0.01). Furthermore, correlation analysis from GSE89632 identified a significant positive correlation between YB1 and INHBE (Figure 3G, *P* < 0.01).

### YB1 knockdown suppressed the expression of INHBE

**Figure 4.**
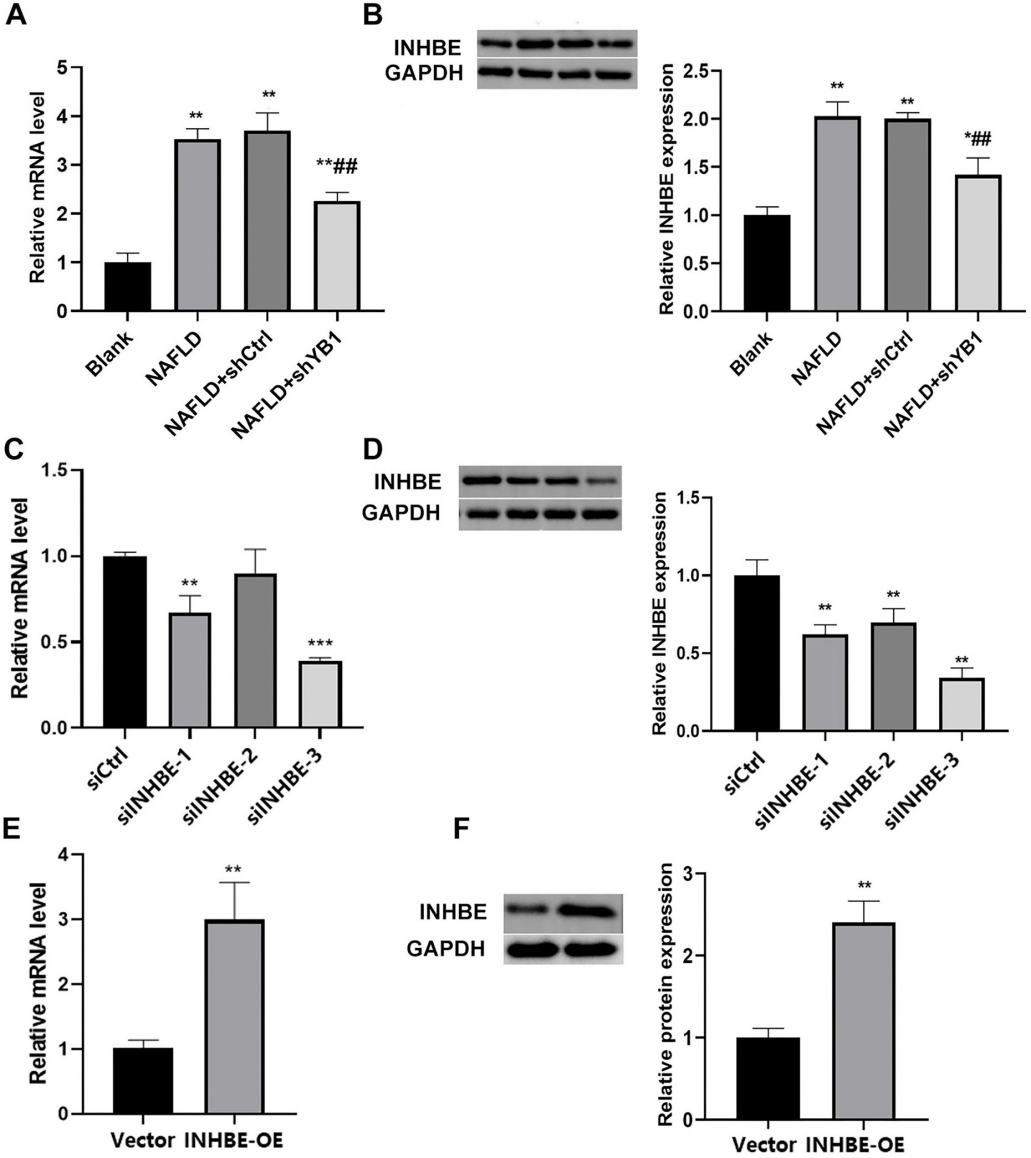
**YB1 knockdown suppressed the expression of INHBE.** (A) QRT-PCR was used to detect the expression of INHBE after YB1 knockdown; (B) Western blot was used to detect the expression of INHBE after YB1 knockdown; (C) Three siRNAs were designed for INHBE, and the expression of INHBE mRNA was detected by QRT-PCR; (D) Western blot was used to detect the expression of INHBE protein in after three siRNAs transfected; (E) QRT-PCR was used to detect the expression of INHBE mRNA after transfected with overexpression plasmid; (F) Western blot was used to detect the expression of INHBE after transfected with overexpression plasmid. Compared to the blank group, *P < 0.05, **P < 0.01, compared to the NAFLD group or NAFLD+shCtrl group, ^##^*P* < 0.01. INHBE: Inhibin beta E; TG: Triglyceride; YB1: Y-box binding protein-1; NAFLD: Nonalcoholic fatty liver disease; QRT-PCR: Quantitative real-time PCR.

To explore the relationship between YB1 and INHBE, we assessed INHBE expression following YB1 knockdown. QRT-PCR and Western blot analyses revealed that INHBE expression was elevated in the NAFLD and NAFLD+shCtrl groups compared to the Blank group. However, INHBE expression was significantly reduced in the NAFLD+shYB1 group relative to the NAFLD and NAFLD+shCtrl groups (Figure 4A and 4B, *P* < 0.05). Among the three siRNAs designed to target INHBE, si-INHBE3 was the most effective in reducing INHBE expression (Figure 4C and 4D, *P* < 0.05). Furthermore, INHBE expression was significantly increased following transfection with an INHBE overexpression plasmid (Figure 4E and 4F, *P* < 0.05).

### YB1 knockdown promoted the lipid metabolism in the NAFLD model cells via INHBE

**Figure 5.**
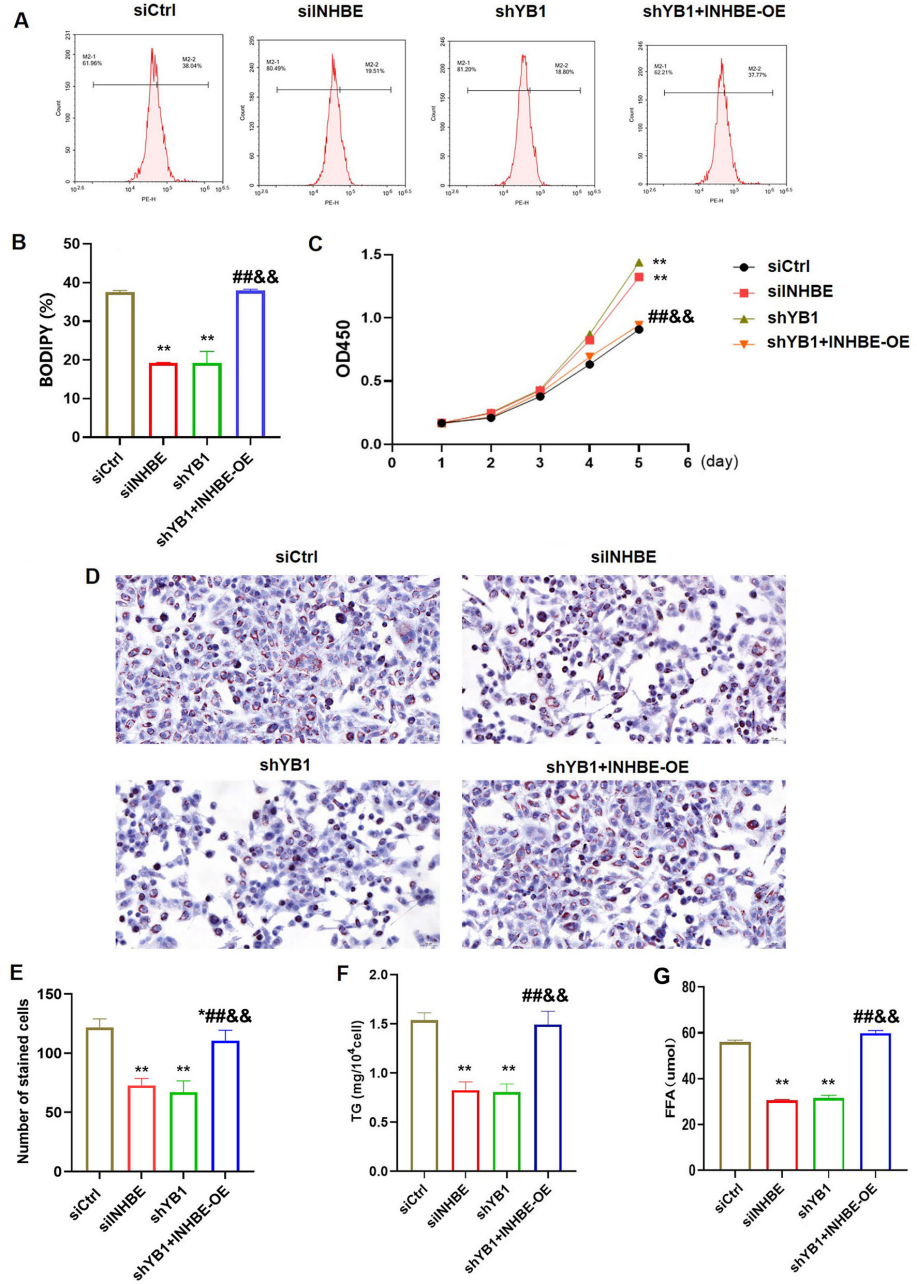
**YB1 knockdown promoted the lipid metabolism in the LO2 cells treatment with PA via INHBE.** (A) Flow cytometry was utilized to detect the fluorescence intensity stained with BODIPY 493/503; (B) Bar graph reflected the percentage of the fluorescence intensity; (C) CCK8 assay was used to examine the LO2 cells viability in different groups; (D) Oil red O staining for lipids in different groups; (E) The cell numbers of oil red O staining was quantified by Image J software; (F) The content of TG was examined in different groups; (G) The content of FFA was examined in different groups. Compared to the siCtrl group, *P < 0.05, **P < 0.01; compared to the siINHBE group, ^##^*P* < 0.01; compared to the shYB1 group, ^&&^*P* < 0.01. INHBE: Inhibin beta E; TG: Triglyceride; YB1: Y-box binding protein-1; PA: Palmitic acid; FFA: Free fatty acid.

To investigate the effects of YB1 and INHBE on lipid metabolism, NAFLD model cells were divided into four groups: siCtrl, siINHBE, shYB1, and shYB1+INHBE-OE. The results showed that knockdown of INHBE and YB1 significantly reduced the fluorescence intensity of BODIPY 493/503, indicating decreased lipid accumulation (Figure 5A and 5B, *P* < 0.01). Moreover, cell viability improved in the siINHBE and shYB1 groups (Figure 5C, *P* < 0.01). Oil Red O staining confirmed a reduction in lipid accumulation following INHBE and YB1 knockdown, specifically demonstrated by a decrease in the number of stained cells (Figure 5D and 5E). Importantly, the levels of TG and FFA were significantly reduced in the siINHBE and shYB1 groups (Figure 5F and 5G, *P* < 0.01). However, INHBE overexpression reversed the beneficial effects of YB1 knockdown.

### YB1 knockdown alleviated the oxidative stress in the NAFLD model cells via INHBE

**Figure 6.**
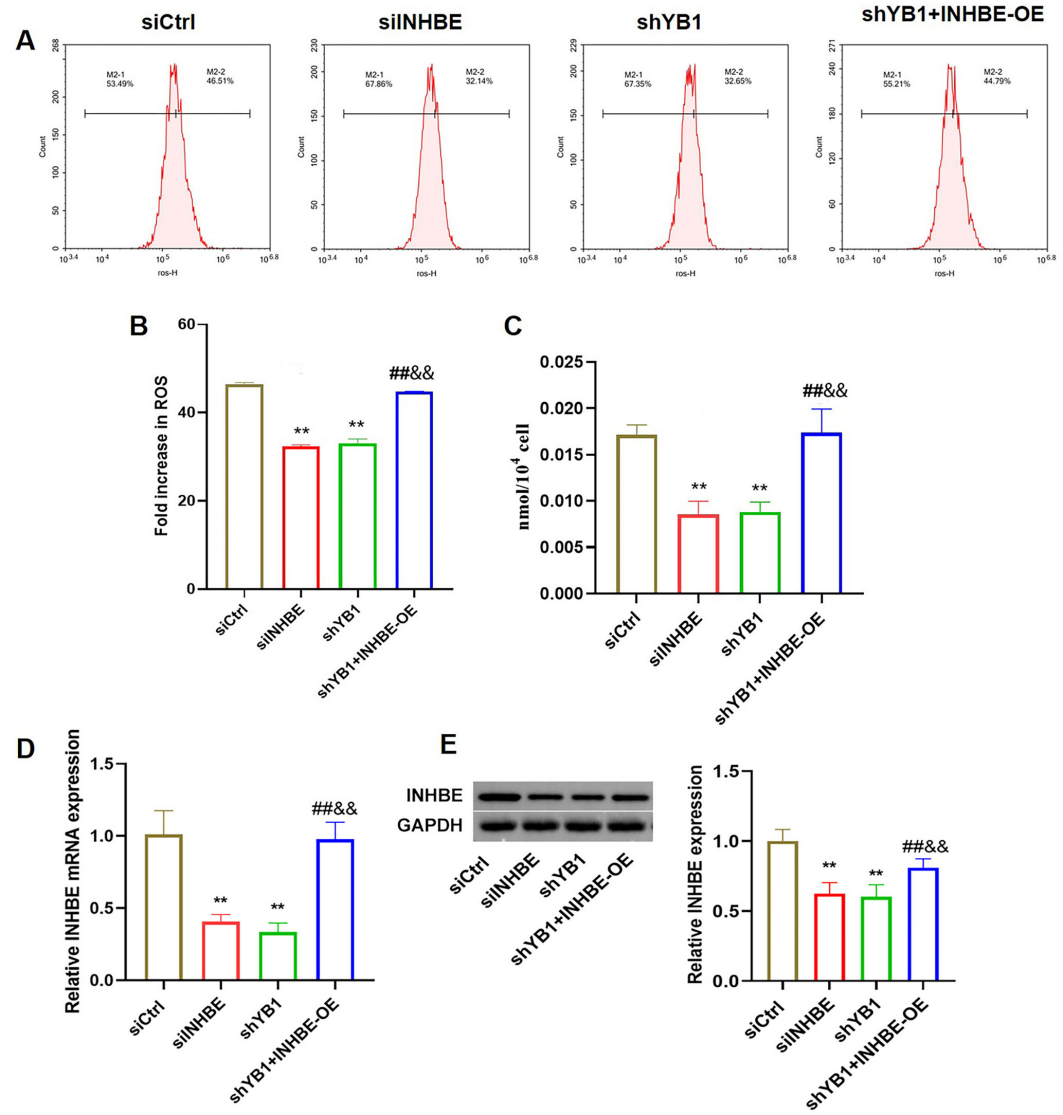
**YB1 knockdown suppressed the oxidative stress in the NAFLD model cells via INHBE.** (A) Flow cytometry was utilized to detect the fluorescence intensity of ROS; (B) Bar graph reflected the percentage of the fluorescence intensity; (C) The content of MDA was examined in different groups; (D) QRT-PCR was used to detect the expression of INHBE mRNA in different groups; (E) Western blot was used to detect the expression of INHBE in different groups. Compared to the siCtrl group, **P < 0.01; compared to the siINHBE group, ^##^*P* < 0.01; compared to the shYB1 group, ^&&^*P* < 0.01. INHBE: Inhibin beta E; NAFLD: Nonalcoholic fatty liver disease; YB1: Y-box binding protein-1; ROS: Reactive oxygen species; MDA: Malondialdehyde; QRT-PCR: Quantitative real-time PCR.

We next analyzed ROS levels and MDA content in the NAFLD model cells. Both ROS fluorescence intensity and MDA levels were significantly decreased in the siINHBE and shYB1 groups compared to the siCtrl group. In contrast, these levels were markedly elevated in the shYB1+INHBE-OE group compared to the siINHBE and shYB1 groups (Figure 6A–6C, *P* < 0.01). Additionally, QRT-PCR and western blot analyses demonstrated that INHBE expression was downregulated in the siINHBE and shYB1 groups, while it was upregulated in the shYB1+INHBE-OE group (Figure 6D and 6E, *P* < 0.01).

### The role of INHBE in NAFLD model cells is linked to the TGF-β signaling pathway

**Figure 7.**
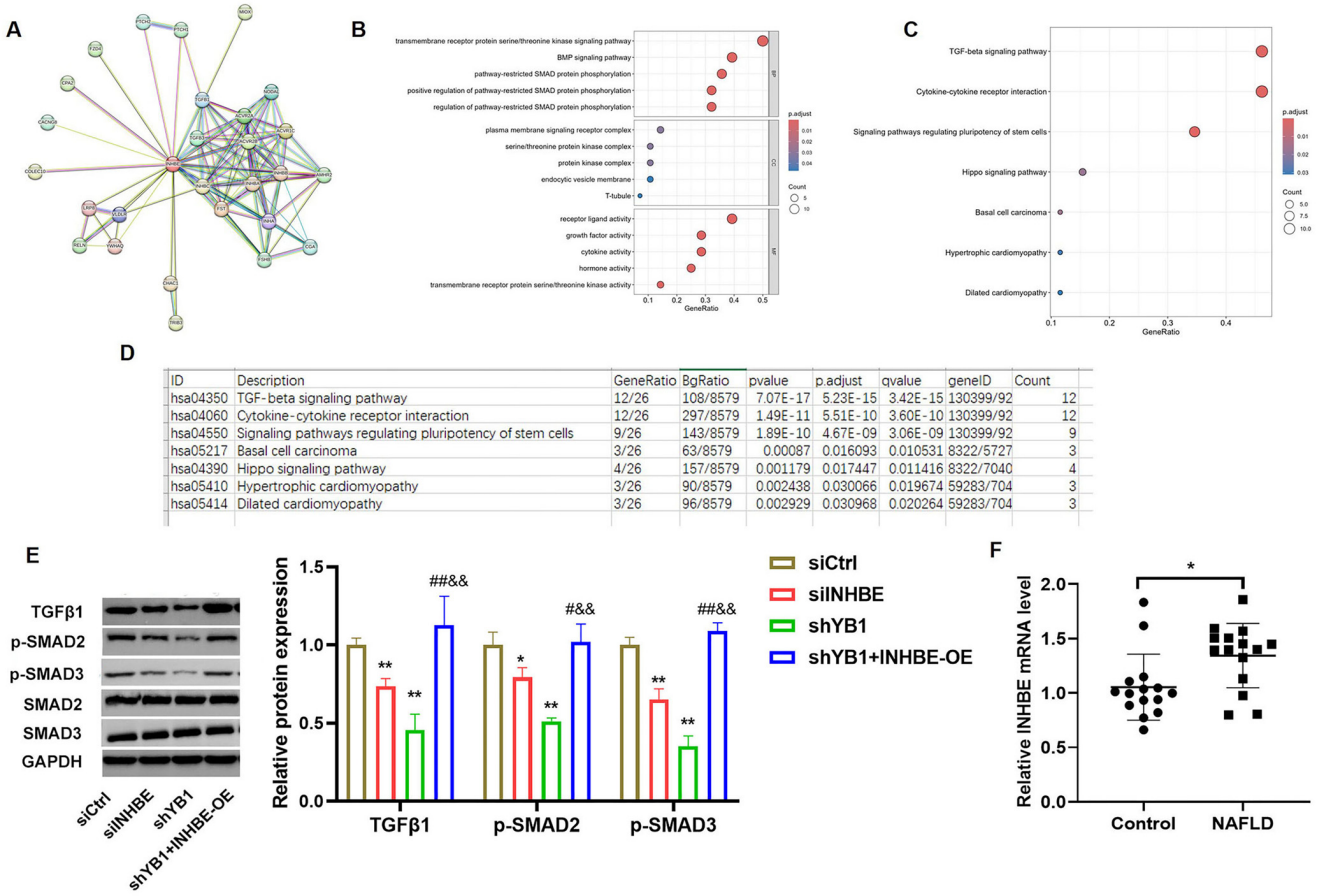
**The underlying mechanism of INHBE in the NAFLD model cells was related to TGF-β signaling pathway.** (A) PPI network and hub genes; (B) Hub genes were analyzed by GO enrichment analysis; (C) Hub genes were analyzed by KEGG enrichment analysis; (D) The related signaling pathways from GO and KEGG were analyzed; (E) Western blot was used to detect the expression of TGFβ1, p-Smad2, p-Smad3, Smad2, and Smad3 in different groups. Compared to the siCtrl group, *P < 0.05, **P < 0.01; compared to the siINHBE group, ^##^*P* < 0.01; compared to the shYB1 group, ^&&^*P* < 0.01; (F) QRT-PCR was used to detect the expression of INHBE mRNA in serum samples. Compared to the Control group, *P < 0.05. INHBE: Inhibin beta E; NAFLD: Nonalcoholic fatty liver disease; PPI: Protein–protein interaction; GO: Gene Ontology; KEGG: Kyoto Encyclopedia of Genes and Genomes; QRT-PCR: Quantitative real-time PCR.

A PPI network identified 27 genes associated with INHBE (Figure 7A). GO and KEGG enrichment analyses revealed that these target genes were primarily involved in the TGF-β and Hippo signaling pathways (Figure 7B–7D). Western blot analysis further demonstrated that the levels of TGF-β1, p-Smad2, and p-Smad3 were decreased in the siINHBE and shYB1 groups but were elevated in the shYB1+INHBE-OE group. Notably, these levels were significantly higher in the shYB1+INHBE-OE group compared to the siINHBE and shYB1 groups (Figure 7E, *P* < 0.05). Additionally, we observed that INHBE mRNA expression was significantly increased in the serum samples of NAFLD patients compared to those of the control group (Figure 7F, *P* < 0.05).

## Discussion

The development of NAFLD is typically slow and insidious, with most cases presenting no obvious symptoms. Despite this, NAFLD poses a significant risk of progressing to cirrhosis and even hepatocellular carcinoma [16]. Previous studies have demonstrated the role of YB1 in promoting tumorigenesis and metastasis in hepatocellular carcinoma [17, 18]. However, the specific role of YB1 in NAFLD remains unclear. In this study, we investigated the effects of YB1 in a NAFLD cell model by knocking down YB1 and analyzing DEGs through transcriptomic analysis. Following YB1 knockdown, we identified 190 upregulated and 608 downregulated genes. Among these, five genes—EGR1, GDF15, INHBE, NUPR1, and FOSB—were identified as key hub genes. EGR1 has been shown to regulate numerous pathways involved in cell proliferation [19]. GDF15, also known as macrophage inhibitory cytokine-1 (MIC-1), is a protein encoded by the GDF15 gene [20]. INHBE has been associated with fat distribution and identified as a circulating growth factor of the activin family that is highly expressed in hepatocytes [21]. NUPR1, a multifunctional transcriptional regulator, is also referred to as COM1 due to its expression in metastatic cancer cells [22]. The FOS gene family, including its members FOS, FOSB, FOSL1, and FOSL2, has been implicated in regulating cell proliferation, differentiation, and transformation [23]. To prioritize a hub gene for further analysis, we examined the expression levels of EGR1, GDF15, INHBE, NUPR1, and FOSB in NAFLD cells. Our results revealed that the expression levels of EGR1, GDF15, NUPR1, and FOSB were significantly reduced, whereas INHBE was markedly elevated. This study confirmed that INHBE expression is elevated in NAFLD cells, but it is reduced following YB1 knockdown, highlighting its important role in NAFLD pathogenesis.

Hepatic lipid accumulation is a hallmark of NAFLD [24]. Using BODIPY 493/503, a widely employed fluorescent probe, we assessed lipid droplet formation in our model [25]. Knockdown of INHBE and YB1 resulted in a significant reduction in lipid droplet formation, as evidenced by a decreased percentage of BODIPY 493/503-positive cells. Additionally, INHBE and YB1 knockdown improved cell viability, reduced lipid accumulation, and lowered TG and FFA levels. Excessive TG accumulation, driven by increased FFA uptake and hepatic lipogenesis, is a key factor in NAFLD development [26, 27]. Our findings indicate that INHBE and YB1 knockdown enhances lipid metabolism, thereby improving NAFLD cell viability [28]. Notably, the effects of YB1 knockdown were reversed by INHBE overexpression, underscoring the interplay between these two factors in regulating lipid metabolism. Previous studies have shown that YB1 regulates liver lipid metabolism via the Wnt/β-catenin signaling pathway [15], and that INHBE and P4HA1 are key hub genes in NAFLD [29]. However, no prior studies have demonstrated a clear correlation between YB1 and INHBE in the context of NAFLD.

ROS and MDA are key indicators of oxidative stress in NAFLD [30, 31]. In our study, we found that both ROS and MDA levels were elevated in NAFLD cells but were significantly reduced following INHBE and YB1 knockdown. Conversely, INHBE overexpression increased ROS and MDA levels, suggesting that INHBE can counteract the protective effects of YB1 knockdown against oxidative stress.To investigate the downstream pathways involved, we performed a PPI network analysis, which identified 27 genes associated with INHBE. GO and KEGG enrichment analyses revealed that these target genes are primarily involved in TGF-β signaling [32]. This pathway is mediated through the TGF-β1 type I receptor, which facilitates the phosphorylation and activation of Smad2 and Smad3, leading to transcriptional activity. Western blot analysis further confirmed that the expression levels of TGF-β1, p-Smad2, and p-Smad3 were reduced following INHBE and YB1 knockdown, while these levels were elevated with INHBE overexpression. These findings suggest that the TGF-β signaling pathway plays a critical role in mediating the effects of YB1 and INHBE in NAFLD. Finally, we measured INHBE levels in serum samples from healthy volunteers and NAFLD patients, confirming that INHBE levels were significantly elevated in NAFLD patients. However, this study has limitations. Our findings are based solely on *in vitro* cell models, and further *in vivo* animal studies are necessary to validate these results. Additionally, the clinical relevance of these findings needs to be confirmed with a larger sample size of NAFLD patients.

## Conclusion

In conclusion, our study demonstrates that YB1 knockdown promotes NAFLD cell viability, suppresses lipid metabolism, and reduces ROS and MDA levels, likely through modulation of the TGF-β signaling pathway. These findings offer valuable insights into the therapeutic potential of targeting YB1 and INHBE in NAFLD treatment, while also highlighting novel directions for future research.

## Supplemental data

**Table S1:** All sequences of primers for qRT-PCR

**Gene**	**Primer sequences (5′ to 3′)**	**Size (bp)**
GAPDH	GGAGCGAGATCCCTCCAAAAT	197
	GGCTGTTGTCATACTTCTCATGG	
EGR1	GGTCAGTGGCCTAGTGAGC	149
	GTGCCGCTGAGTAAATGGGA	
FOSB	GCTGCAAGATCCCCTACGAAG	249
	ACGAAGAAGTGTACGAAGGGTT	
GDF15	GACCCTCAGAGTTGCACTCC	75
	GCCTGGTTAGCAGGTCCTC	
NUPR1	CTCTCATCATGCCTATGCCTACT	61
	CCTCCACCTCCTGTAACCAAG	
INHBE	ATCTTCCGATGGGGACCAAG	95
	AGAGTTAAGGTATGCCAGCCC	
YB1	GGGGACAAGAAGGTCATCGC	155
	CGAAGGTACTTCCTGGGGTTA	

YB1: Y-box binding protein-1; INHBE: Inhibin beta E; QRT-PCR: Quantitative real-time PCR.

## Data Availability

The data used to support the findings of this study are available from the corresponding author upon request.
